# Use of a 10.22 m diameter EPB shield: a case study in Beijing subway construction

**DOI:** 10.1186/s40064-016-3672-5

**Published:** 2016-11-24

**Authors:** Xinggao Li, Dajun Yuan, Yuhai Guo, Zhiyong Cai

**Affiliations:** 1 School of Civil Engineering, Beijing Jiaotong University, Beijing, 100044 China; 2 Beijing Municipal Construction Co., Ltd., Beijing, China

**Keywords:** 10.22 m Diameter EPB shield, Spoke-type cutterhead, Soil conditioning, Simultaneous backfilling grouting

## Abstract

**Introduction:**

Beijing subway line 14 includes four stations and approximately 2.8 km of tunnels between the Dongfengbeiqiao and Jingshunlu areas of the city. Due to the surface and underground space limitations of this section, a double-track running tunnel instead of two single-track running tunnels was adopted to connect the two stations. The double-track tunnels were excavated by a 10.22 m diameter earth pressure balance (EPB) shield. It was the first time that an EPB shield more than 10 m in diameter was used in Beijing subway construction.

**Case description:**

The shield, which passes underneath densely built-up areas of the city and is equipped with a spoke-type cutterhead, with balance between the ground pressure and the earth chamber pressure at the tunnel face, is of great importance. Referring to experiences gained in the EPB shield tunneling, attention was paid to the function of soil conditioning and simultaneous backfilling grouting of the shield, and some special designs were considered in manufacturing the machine.

**Discussion and Evaluation:**

In addition to the agitating rods welded to the cutterhead, two independently driven agitators were added to fully mix everything in the earth chamber. Independent pipelines were arranged for injecting different conditioning agents. Indoor tests in combination with field tests were conducted to find suitable additives and injection ratios of the additives, and determine the mix ratio of the two-component grout for simultaneous backfilling grouting. A scheme was employed for simultaneously injecting the bentonite slurry at 8% concentration and the foam liquid at 5% concentration to condition the excavated soil. The cement–sodium silicate grout was adopted to fill the tail void and the injection volume per ring was 14.1–15.3 m^3^.

**Conclusions:**

The performance of the shield and evaluation of the corresponding tunneling technologies are introduced in terms of the shield tunneling induced ground surface settlements. The success of the project is of great significance to Beijing subway construction and underground space utilization. The findings serve as a useful reference for similar projects.

## Background

In the past decade, the subway system has been increased dramatically due to the large population and limited surface spaces in Beijing, which is the capital and one of the most important economic centers of China. For the underground lines in the city, usually subway stations are connected by two running tunnels: one down-line tunnel and one up-line tunnel. Beijing is currently extending its subway network, although the underground space of the city is becoming increasingly more scarce, especially at shallow depths. Therefore, sufficient space to accommodate spaced twin single-track running tunnels does not always exist, and constructing a subway station using conventional methods and technologies can’t be done smoothly due to restrictions caused by surrounding environment. However, these problems can be effectively addressed when a double-track tunnel is adopted instead of twin single-track running tunnels, where the subway stations are formed by expanding the finished double-track tunnel. The construction planning of the project is running tunnel construction oriented rather than station construction oriented. Stations can be planned in advance and constructed when necessary. Moreover, in a double-track tunnel, a crossover can be easily realized. Double-track tunnels are constructed using the large diameter earth pressure balanced (EPB) shield tunneling method because of the many advantages it offers over conventional methods. Thus, the smooth and rapid construction of the shield tunnel is of great importance for the on-schedule completion of the project. This is explained by use a 10.22 m diameter EPB shield to build the double-track tunnels on Line 14 of the Beijing subway.

It was the first time that an EPB shield more than 10 m in diameter was used in Beijing subway tunnel construction. The main focus was on the equipment required for soil conditioning and simultaneous backfilling grouting of the large diameter EPB shield when designing the shield in the initial stage of the project. During the period of constructing the double-track tunnels, emphasis was placed on determining the key driving parameters, such as earth chamber pressure and advance rate. The shield tunneling was completed as planned. This paper presents the special designs and performance of the EPB shield in terms of the maximum settlements of the ground surface.

## Project overview

**Fig. 1:**
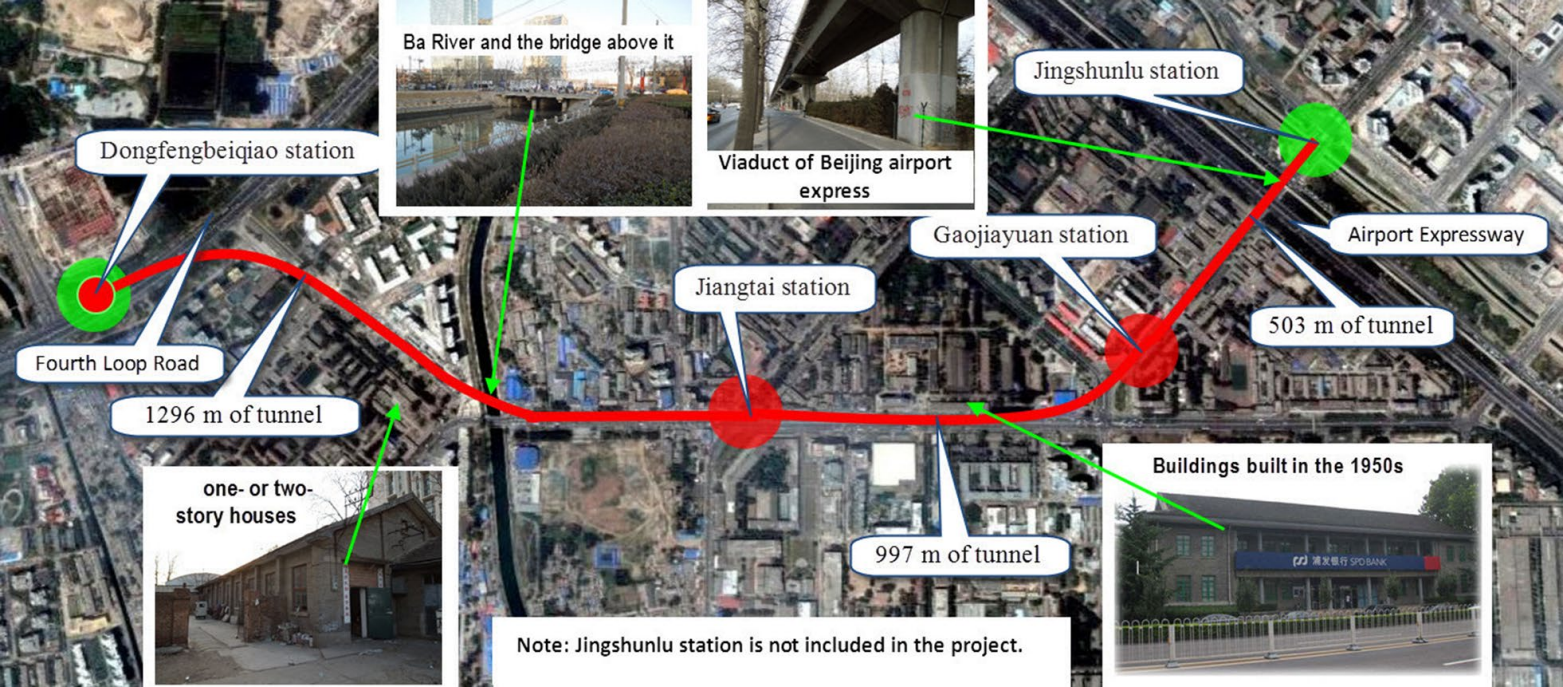
Beijing subway line 14 route between Dongfengbeiqiao station and Jingshunzhan station

The project includes three stations (Dongfengbeiqiao station 298.7 m in length, Jiangtai station 166 m in length and Gaojiayuan station 179 m in length) and approximately 2507.9 m of double-track tunnels on Beijing subway Line 14, as shown in Fig. 1. Instead of two single-track running tunnels, one double-track running tunnel was adopted to connect two neighboring stations due to the limited surface and underground space. A 10.22 m diameter EPB shield was chosen, after overall considerations, to excavate the double track tunnels. To reduce the project duration, the construction plan employed involved completing the continued 3151.6 m long shield driving first, followed by construction of the two stations (Jiangtai and Gaojiayuan stations) by expanding the finished shield tunnel. The enlargement was realized by using a modified PBA method (Liu et al. 2015). The PBA method is a manual excavation method, which is widely used in Beijing to build subway stations.

**Fig. 2:**
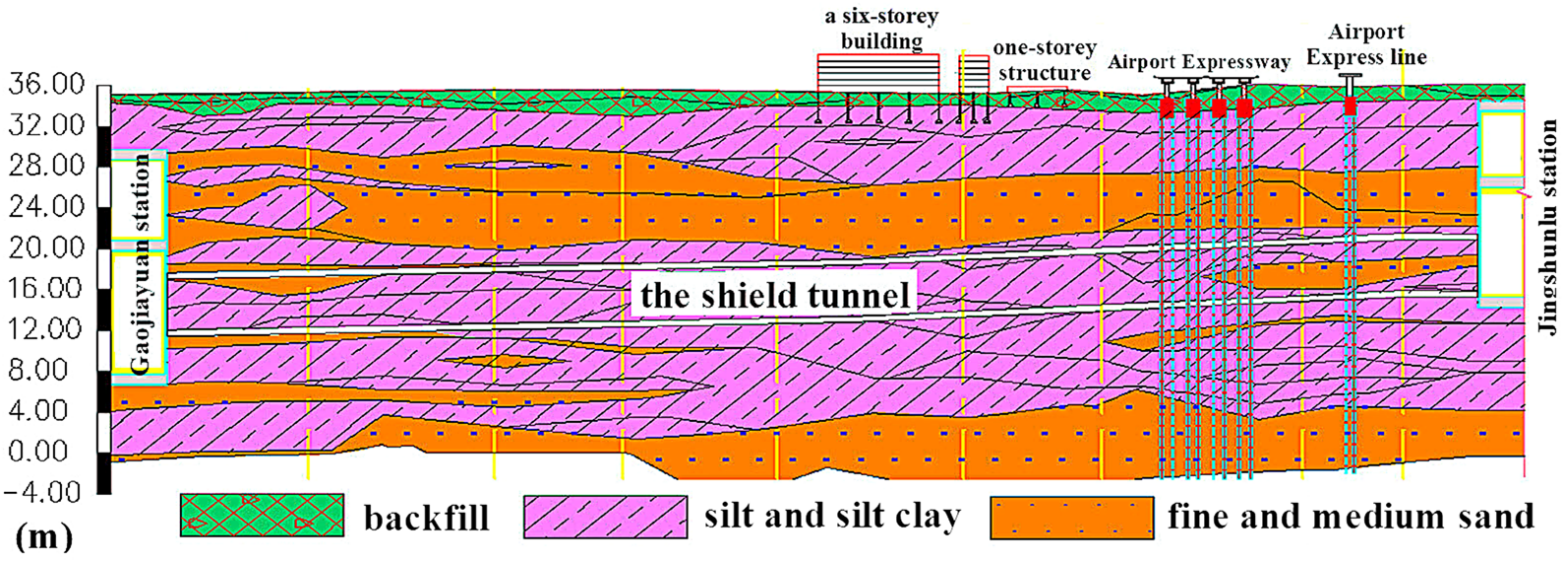
Longitudinal section between Gaojiayuan station and Jingshunlu station

The soils at the job site can be divided into three groups: (1) backfill; (2) silt and silty clay and (3) fine and medium sand, as shown, taking the running tunnel between Gaojiayuan station and Jingshunlu station as an example, in Fig. 2. The soil group properties are listed in Table 1. There are four types of aquifers involved: (1) perched aquifer, (2) unconfined aquifer, (3) confined aquifer I and (4) confined aquifer II, with water tables of 2.98–6.79, 5.98–9.25, 11.70–18.40 and 21.00–27.65 m underneath the surface, respectively. The overburden depth ranges from 11.3 to 21.2 m. Two elements concerning the tunnel alignment are the minimum curve radius of 350 m and the maximum slope of 27‰. The soils of sand, rich in ground water, the shallow overburden, and the small curve radius will together contribute to high surface settlement if not properly addressed.

**Table 1:** Soil group properties

Soil group	Natural density (g/cm^3^)	Dry density (g/cm^3^)	Porosity	Liquid limit (%)	Plastic limit (%)	SPT N-values
Backfill	1.58–1.65	–	–	–	–	–
Silt and silty clay	1.89–2.08	1.43–1.76	0.349–0.476	26.1–40.9	17.6–25.4	–
Fine and medium sand	2.02–2.10	–	–	–	–	23–53

The project was constructed in the densely built-up areas of Beijing. The large diameter shield tunneling influenced the nearby buildings and structures. The shield drove under the main city roads, so the road surface and buried pipelines along the roads were affected. The involved pipelines include the heat supply, gas, water supply and drainage, cable, communication pipelines and so forth. Other risk sources identified are the numerous one- or two-story houses, the low-rise buildings built in the 1950s, Ba River and the bridge above the river, the Fourth Loop Road, the Airport Expressway and the viaduct of Beijing airport express, under which the shield passes, as shown in Fig. 1. The surroundings were sensitive to the shield tunnel construction, which posed significant challenges to the project.

## Selection of shield types and special design of the shield

### Selection of shield types

**Fig. 3:**
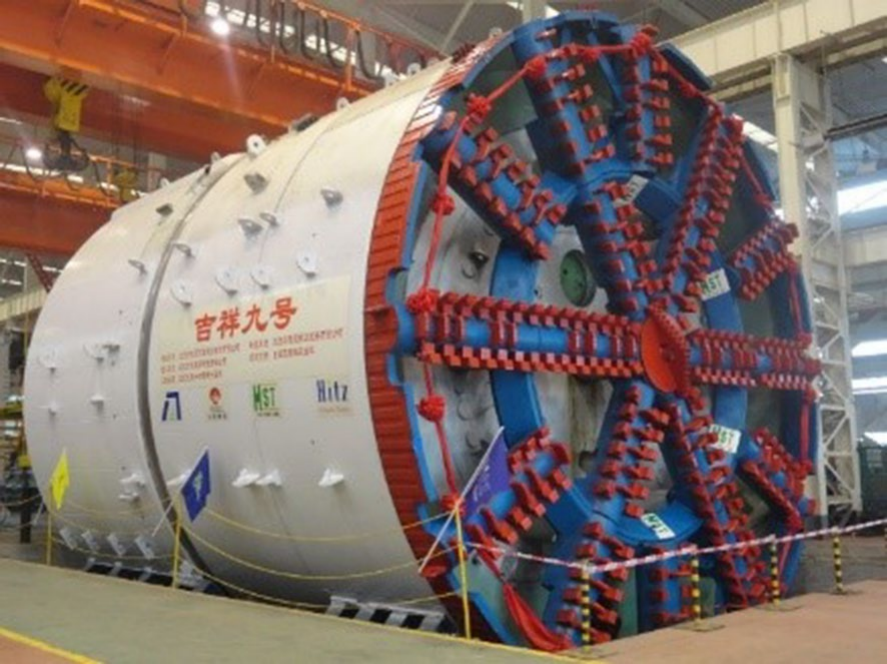
The used 10.22 m diameter EPB shield

**Fig. 4:**
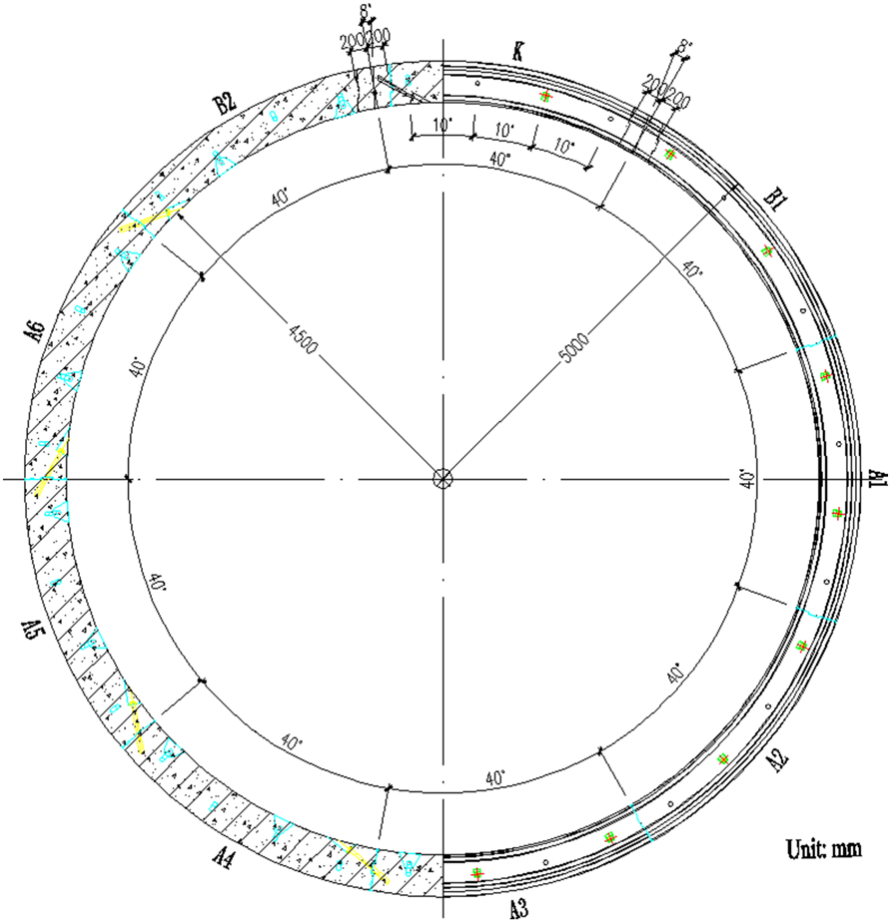
A cross-section of the tunnel

The selection between the EPB- and slurry-shields is influenced by three types of factors: economic, technical and geological, and a thorough and detailed discussion is often required. However, the use of an EPB shield in this project can be justified by two reasons: (1) the accumulated experience and skill with the use of the 6.15 m diameter EPB shields in Beijing area and (2) the lack of sufficient ground surface space for a sophisticated separation plant for the slurry shield. As presented in Fig. 3, the used machine is equipped with a spoke-type cutterhead, with an opening ratio of 65%. Important technical parameters of the shield are summarized in Table 2. In Table 2, the maximum torque coefficient is related to the installed cutterhead torque. The most widely used empirical formula for the installed cutterhead torque of soft ground shield machines is *T* = *α D* ^3^, where *T* is cutterhead torque (kN m), and *D* is excavation diameter of the machine (m) and *α* is the maximum torque coefficient. A cross-section of the tunnel is presented in Fig. 4.

**Table 2:** Important technical parameters of the 10.22 m diameter shield

Items	Parameters
Excavation diameter (mm)	10,220
External diameter (mm)	10,000
Internal diameter (mm)	9000
Segment thickness (mm)	500
Segment ring width (mm)	1800
Configuration of segments	9 (8 + 1 keystone)
Machine length (mm)	11550
Cutterhead rotation speed (r/min)	0–0.68
Advance rate (mm/min)	0–85
Total thrust (kN)	108,000
Maximum torque coefficient (α)	α = 32.2
Working torque (kN m)	22,896–34,344

### Special design of the shield

#### Design of the soil conditioning

 The EPB shield uses the excavated material by the cutting wheel as the support medium to maintain the stability of the cutting face, and the supporting pressure is heavily dependent on the plastic flow of the muck in the earth chamber. In particular, ensuring high plastic flow of the muck matters when operating the 10.22 m diameter shield is important because the excavated volume per meter by the machine is nearly 2.8 times greater than that by the 6.15 m diameter shield. When shield driving in the soils of this project, high fluidity cannot be achieved by filling the excavation chamber with the excavated soil. Moreover, the soil conditioning should take place directly during excavation at the face in front of the cutting wheel to prevent the material sticking to cutting wheels. Besides choosing suitable conditioning agents, the 10.22 m diameter shield machine should be equipped with necessary components to realize the uniform injection of the agents and the full agitation of the agents with the excavated soils. Three aspects of the machine design were involved in the machine type selection stage: (1) devices for injecting bentonite slurry and foam liquid, and corresponding layouts of pipelines, (2) locations and amounts of injection ports, and (3) the agitating device to actively and independently mix the additives with the soils.

**Fig. 5:**
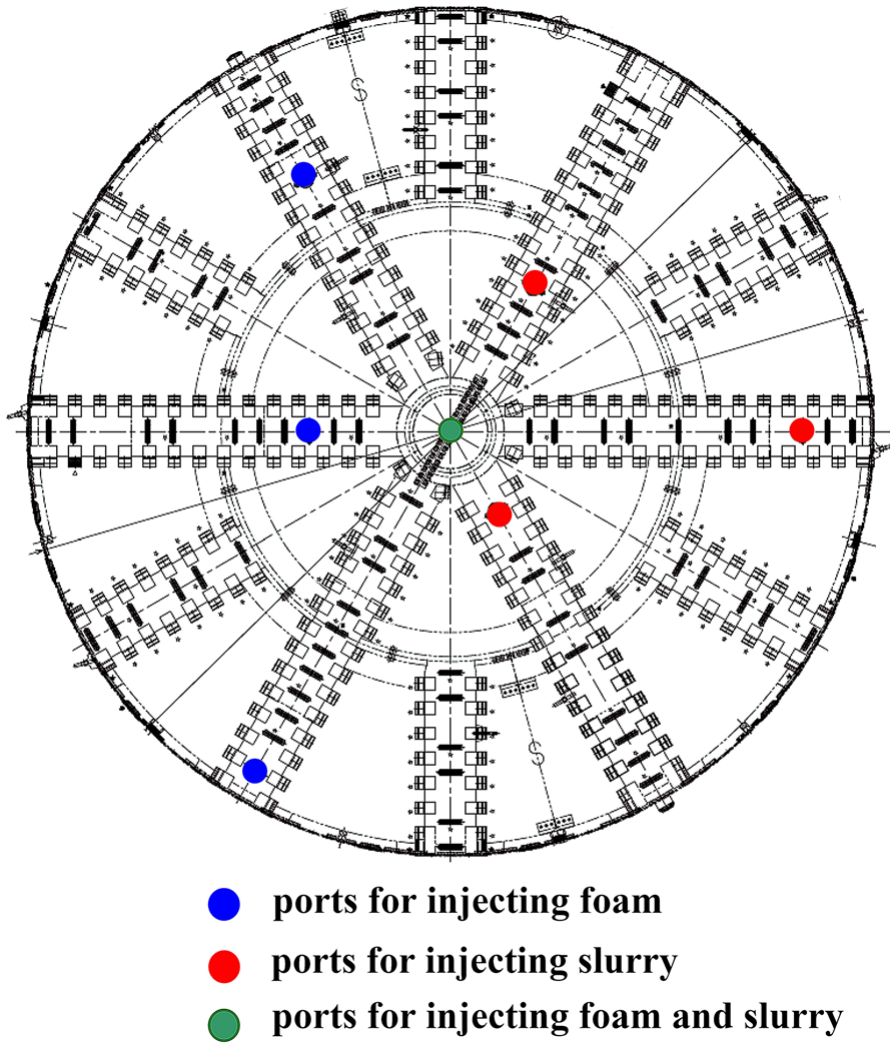
Injection ports at the cutting wheel

Six slurry pumps and six foam pumps were provided for the 10.22 m diameter shield machine after the full argumentation and in depth analysis. The additive injection units consisting of injection pumps and injection nozzles located on the cutterhead and in the chamber were determined according to the soil conditions and the machine structure. Twelve additive injection ports were provided at the cutting wheel, bulkhead and crew conveyor of the machine. The injection ports at the cutting wheel are shown in Fig. 5.

**Fig. 6:**
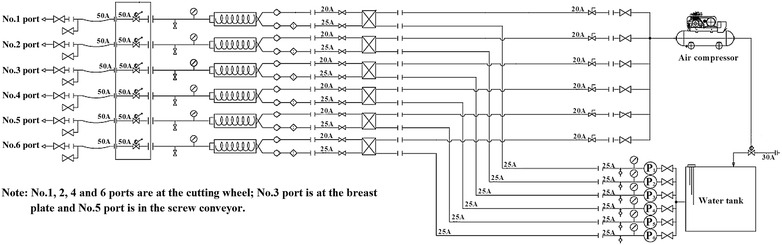
Diagram of the pipeline layout employed for injecting the foam liquid

**Fig. 7:**
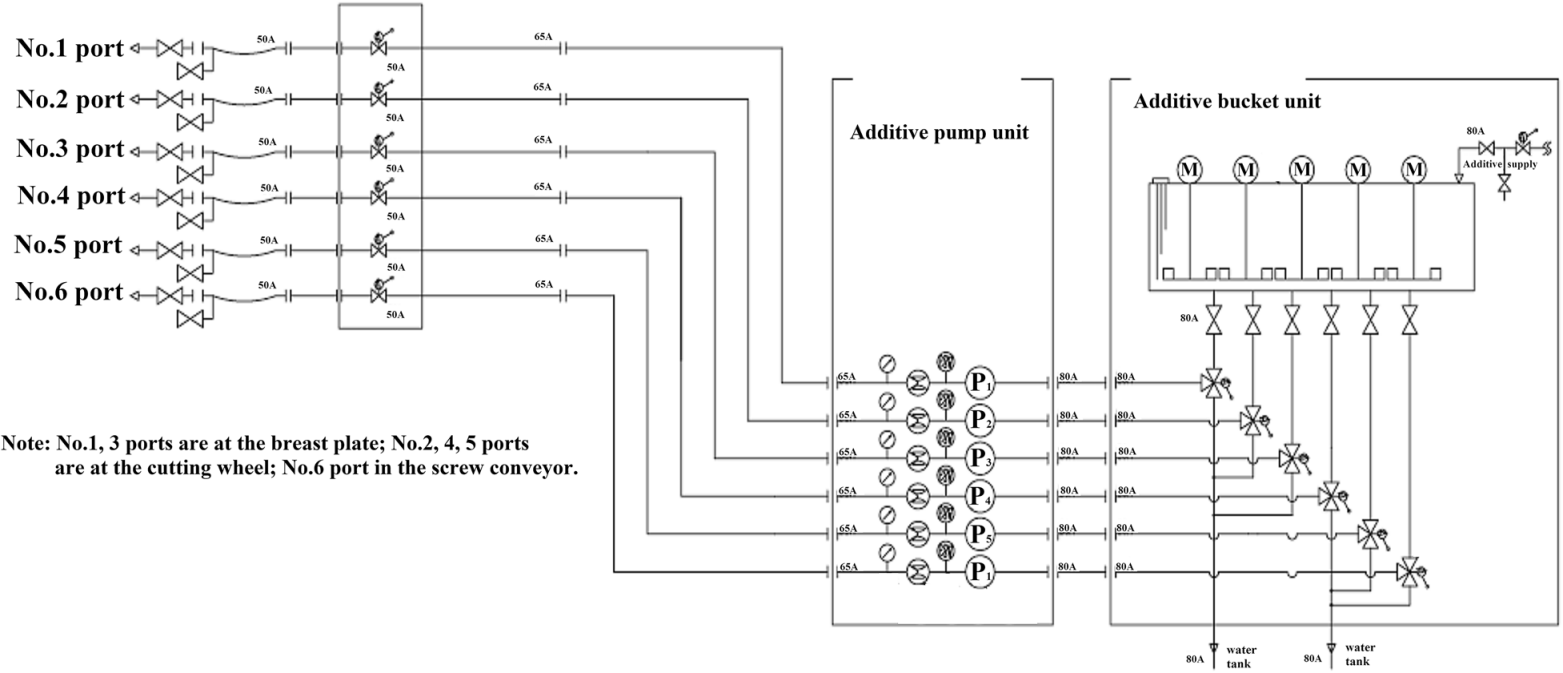
Diagram of the pipeline layout employed for injecting the bentonite slurry

 Independent pipelines for injecting different conditioning agents were arranged as shown in Figs. 6 and 7.

**Fig. 8:**
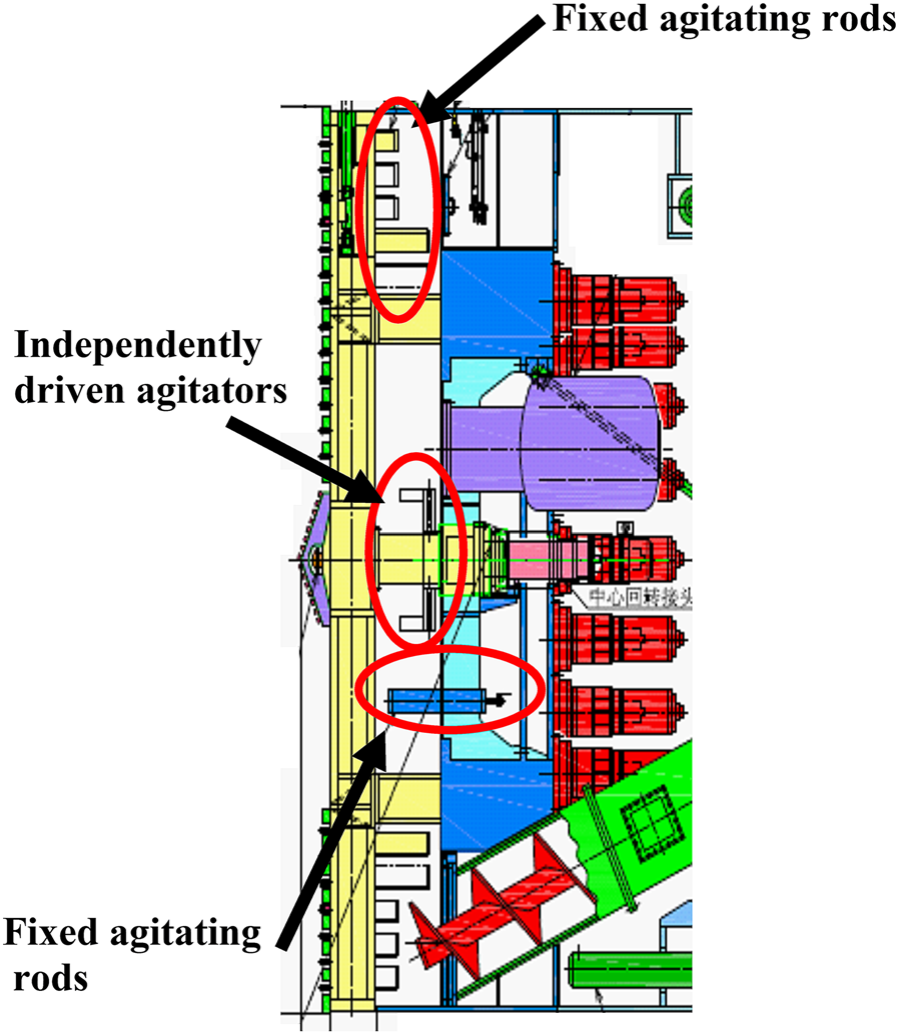
Fixed agitating rods and independently driven agitators

**Fig. 9:**
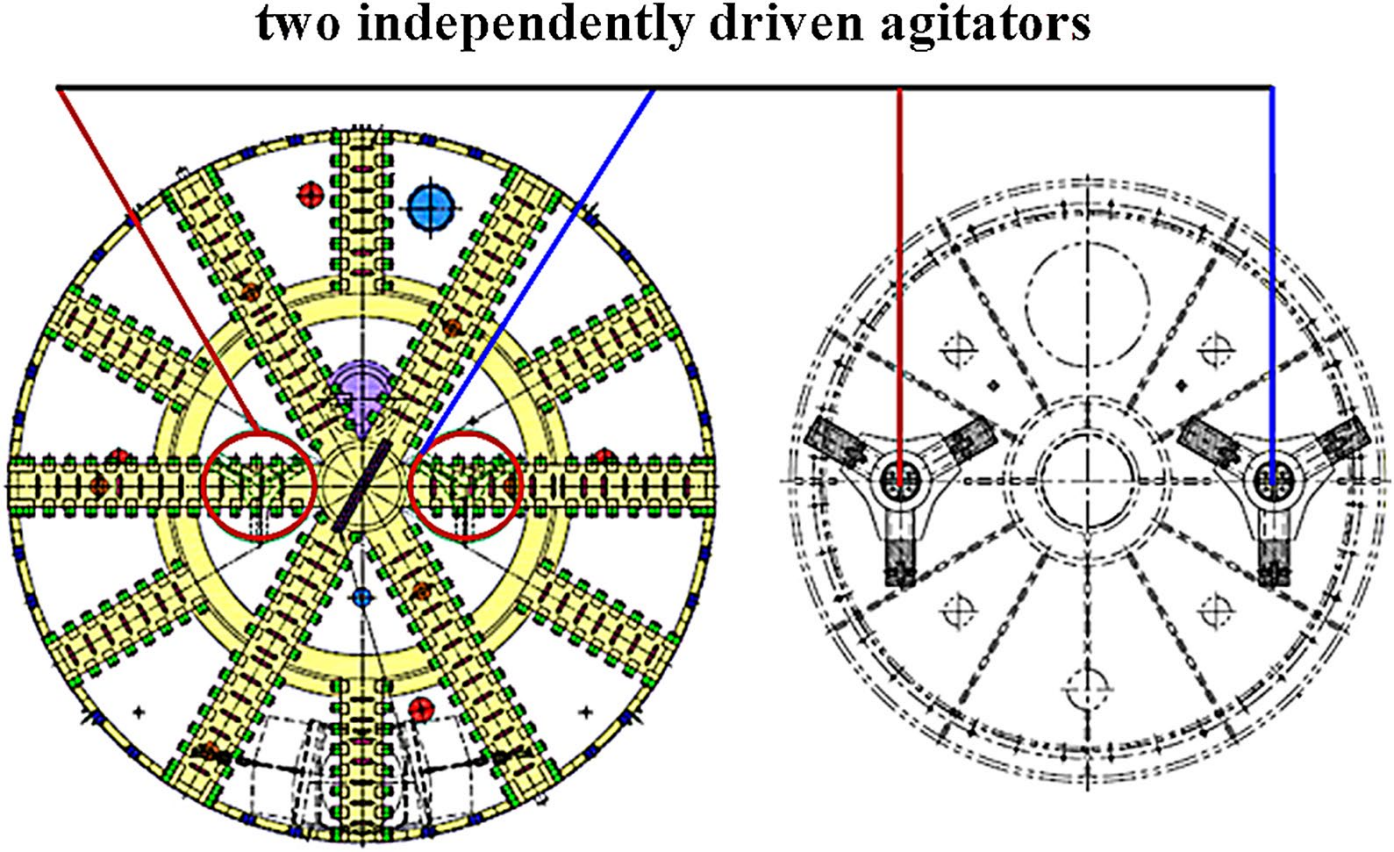
The independently driven agitators located behind the cutting wheel

In addition to the agitating rods attached to the cutterhead and the fixed rods mounted on the bulkhead (see Fig. 8), two independently driven agitators (see Fig. 9), symmetrically arranged on both sides of and near the cutter head center, were employed to enhance the mixing of the excavated soil and additives and to prevent any blocking of the cutting wheel or blockage of the excavation chambers in areas with a low pressure gradient. Full mixing of the excavated soil with the injected additives ensured plastic flow of the muck in the shield driving process.

At the onset of the shield tunneling, the comparison on the cutterhead torque between on and off rotation of the agitators was done to test the functioning of the agitators when the machine at a low advance rate of about 10 mm/min. An about 20% increase in the torque resulted when switching off the agitators. It was expected that the agitators would play a more important role when shield advancing at the normal rate of 50 mm/min.

#### Grouting equipment

**Fig. 10:**
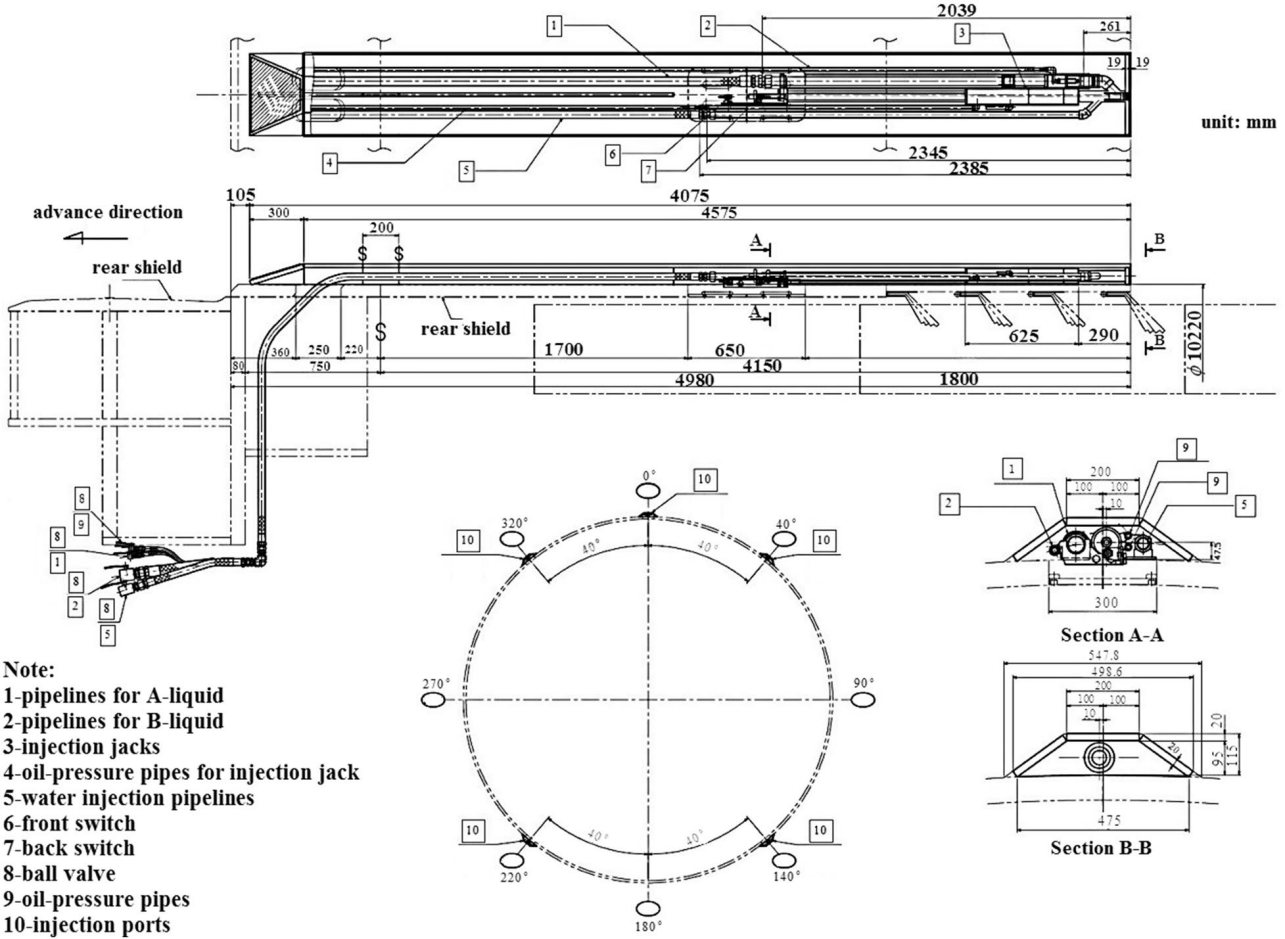
Layout and makeup of the simultaneous backfilling grouting system

In view of the efficacy of confining the ground movements of backfilling using the two-component grouts, two-component system injection is progressively substituting the traditional use of cementitious mortars (Peila et al. 2011). The equipment for the two-component system was required for the 10.22 m diameter shield. Five sets of simultaneous backfilling grouting systems were arranged at five positions along the circumference of the 10.22 m diameter shield, as shown in Fig. 10. The system consisted of injection pipelines for A-liquid and/or B-liquid, injection jacks, oil-pressure pipes for the injection jack, water injection pipelines, a front switch, a back switch, a ball valve, oil-pressure pipes and injection ports. Additionally, three pumps for the A-liquid, three pumps for the B-liquid, two water pumps, and corresponding storage tanks, pipelines were provided.

## Key shield tunneling technologies

Since the shield passed underneath the densely built-up areas of the city, influences of the shield tunneling were strictly controlled within the accepted levels. In addition, it was critical that the shield tunnel construction was completed as scheduled. Any delay to cope with the influences could slow down or obstruct shield machine operations if not properly handled. To allow for safe and accurate tunnel excavation at a normal speed and without adverse effects on the surroundings, two shield tunneling technologies should be highlighted: soil conditioning and simultaneous backfilling grouting.

### Soil conditioning

To use the removed soil from the face as a support medium, it should possess the following properties: (1) high plastic ductility, (2) pasty to soft consistency, (3) low internal friction, and (4) low water permeability (Maidl et al. 2012). When using the 6.15 m diameter EPB shields in Beijing, the slump of the muck is often employed to evaluate the properties of the conditioned soils, and the slumps in the range of 100–150 mm (vane shear strength of 0–5 kPa) are appropriate to serve as the support medium to counteract soil and water pressure at the cutting face, which is the experience gained from the past shield tunneling in Beijing. The soils encountered in this shield driving have a slump of approximately 55 mm in their natural state, hence most have to be conditioned. Soil conditioning can be conducted by the addition of either bentonite or foam, or of both. To further verify suitable conditioning agents and determine the injection volume, a combination of indoor experiments and in situ experiments was used in the project.

#### Indoor tests

In indoor tests, the conditioning effects was tested by injecting bentonite and foam individually, and injecting both of them simultaneously. In the experiments, sodium bentonite was adopted with a colloid index of more than 400 ml/15 g and an expansion rate of more than 25 ml/g. The conditioning effects varies with the bentonite slurry concentration (Concn) and the bentonite injection ratio (BIR). Field experience in Beijing has indicated that a bentonite slurry with a Concn of 8% achieves optimal results. This bentonite slurry was used in the indoor experiments to condition the silt with different BIRs. The measured slumps of the samples are given in Table 3. The properties of the foaming agent used are listed in Table 4. The slumps were recorded for the tested silt samples with the various foam injection ratios (FIRs) and foam expansion ratios (FERs), as presented in Table 5. In Table 5, the foam height was measured as the Ross–Miles method (Al-Sabagh et al. 2003): 200 ml of a solution of surfactant contained in a pipette of specified dimensions with a 2.9 mm internal diameter, from the orifice 90 cm are allowed to fall onto 50 ml of same solution contained in a cylindrical vessel maintained at a given temperature by means of a water jacket. The height of the foam produced in the cylindrical vessel was read immediately after all the solution had run out of the pipette (initial foam height) and again after a given amount of time (generally 5 min). Properties of the soil samples are listed in Table 6.

**Table 3:** Measured slumps of the samples with different BIRs

Concn of the bentonite slurry (%)	Water (kg)	Bentonite (kg)	Density (g/cm^3^)	Viscosity (s)	BIR (%)	Slump (mm)
8	1000	89	1.04	27	15	78
					10	72
					5	65

**Table 4:** Properties of the foaming agent

Items	Index
Appearance	Colorless transparent liquid
Water solubility	Completely soluble
Density, 25 °C	1.01 ± 0.02 g/cm^3^
PH value	7–8
Foaming capacity (Ross–Miles)	≥220 mm

**Table 5:** Measured slumps of the samples with different FIRs and FERs

Concn of the foam liquid (%)	Mass of the foam liquid (kg)	Water (kg)	FER	FIR (%)	Density (g/cm^3^)	Slump (mm)
5	50	950	10	15	1.96	82
				20	1.92	85
			12	15	1.93	84
				20	1.87	87
			15	15	1.89	72
				20	1.81	72

**Table 6:** Properties of the silt samples

Soil sample	Natural density (g/cm^3^)	Dry density (g/cm^3^)	Porosity	Liquid limit (%)	Plastic limit (%)
Silt	2.08	1.76	0.349	26.1	17.6

The scheme involved the simultaneous injection of the bentonite slurry and foam liquid to condition the excavated soil. Based on construction experiences in similar geologic conditions, three recipes were tested: (1) 8% bentonite slurry with 5% foam liquid, (2) 6% bentonite slurry with 7% foam liquid and (3) 10% bentonite slurry with 3% foam liquid. In the indoor tests, various injection ratios were used for the soil samples. Recorded slumps of the samples are listed in Tables 7, 8 and 9. It was found that the first recipe of the 8% bentonite slurry with the 5% foam liquid achieved the best results.

**Table 7:** Recorded slumps of the samples using the first recipe (unit:mm)

BIR of the 8% bentonite slurry (%)	4.0	6.0	8.0
FIR of the foam liquid (5% of Concn and 10 of FER) (%)			
10	105	140	179
15	124	166	199
30	151	189	215
FIR of the foam liquid (5% of Concn and 12 of FER) (%)			
10	102	*137*	175
15	117	*165*	192
30	143	*185*	211
FIR of the foam liquid (5% of Concn and 15 of FER) (%)			
10	98	130	164
15	103	138	176
30	118	155	190

The italics in the tables present the results with the most appropriate FER of the foam liquid

**Table 8:** Recorded slumps of the samples using the second recipe (unit:mm)

BIR of the 6% bentonite slurry (%)	4.0	6.0	8.0
FIR of the foam liquid (7% of Concn and 10 of FER) (%)			
10	96	127	158
15	108	142	174
30	133	165	199
FIR of the foam liquid (7% of Concn and 12 of FER) (%)			
10	96	*128*	155
15	107	*140*	170
30	132	*163*	192
FIR of the foam liquid (7% of Concn and 15 of FER) (%)			
10	91	122	150
15	96	128	160
30	108	142	175

The italics in the tables present the results with the most appropriate FER of the foam liquid

**Table 9:** Recorded slumps of the samples using the third recipe (unit:mm)

BIR of the 10% bentonite slurry (%)	4.0	6.0	8.0
FIR of the foam liquid (3% of Concn and 10 of FER) (%)			
10	77	110	153
15	95	135	170
30	120	161	189
FIR of the foam liquid (3% of Concn and 12 of FER) (%)			
10	70	*109*	144
15	89	*129*	163
30	111	*156*	185
FIR of the foam liquid (3% of Concn and 15 of FER) (%)			
10	65	102	135
15	72	110	147
30	85	125	164

The italics in the tables present the results with the most appropriate FER of the foam liquid

#### Field tests

**Fig. 11:**
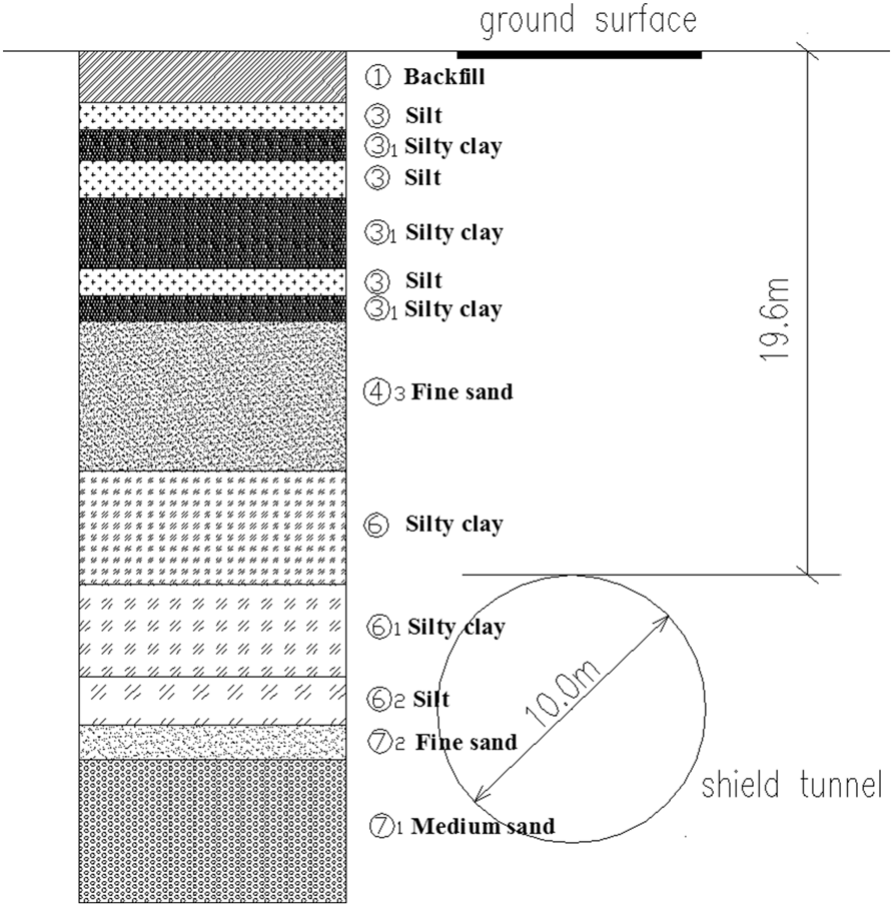
Soils at the test sections

For construction of the shield tunnels in Beijing, which were a little more than 6 m in outer diameter, slumps of approximately 130 mm of the conditioned soils were sufficient. In the initial construction stage of the project, it was attempted to use the existing conditioning scheme in the shield driving. However, operation of the 10.22 m diameter shield was difficult. The earth chamber pressure, cutterhead torque and total thrust fluctuated widely, and large clods of earth existed, resulting in obvious ground surface settlement and a wider settlement trough. It was absolutely necessary to perform field tests to further optimize the conditioning scheme based on the indoor experiments. Considering the ease of operation and engineering economy, the scheme of simultaneously injecting 8% bentonite slurry and 5% foam liquid was adopted in the field tests based on the above findings. Through the field tests, the differences in conditions between the indoor tests and the construction were justified and the actual effects of the conditioning agent and the injection ratios were further verified. The test sections is given in Fig. 11 and properties of the soils are presented in Table 10.

**Fig. 12:**
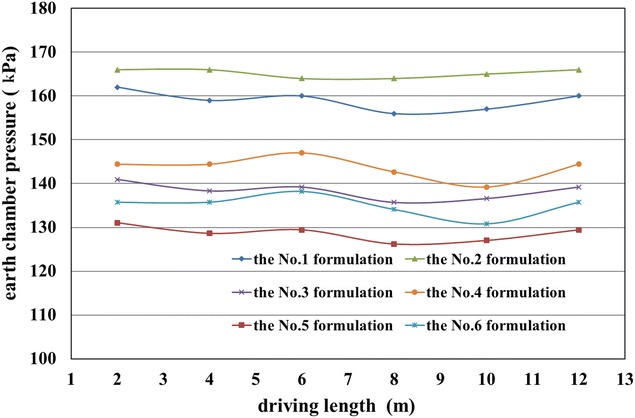
Recorded earth chamber pressure

**Table 10:** Properties of the soils at the test sections

Name	Natural density (g/cm^3^)	Dry density (g/cm^3^)	Porosity	Liquid limit (%)	Plastic limit (%)	SPT N-values
Backfill ①	1.65	–	–	–	–	–
Silt ③	2.02	1.67	0.369	26.3	19.2	–
Silty clay ③_1_	1.90	1.46	0.463	36.9	24.3	–
Fine sand ④_3_	2.02	–	–	–	–	31
Silty clay ⑥	2.03	1.66	0.389	31.8	19.6	–
Silty clay ⑥_1_	1.89	1.43	0.476	40.9	24.9	–
Silt ⑥_2_	2.08	1.76	0.349	26.1	17.6	–
Medium sand ⑦_1_	2.10	–	–	–	–	53
Fine sand ⑦_2_	2.05	–	–	–	–	44

According to the results presented in Tables 7, 8 and 9, six formulations of foam liquid and bentonite slurry were used in the in situ tests:

1. The No. 1 formulation: 15% of FIR (Concn of 5%, FER of 12) and 6% of BIR (Concn of 8%);
2. The No. 2 formulation: 30% of FIR (Concn of 5%, FER of 12) and 6% of BIR (Concn of 8%);
3. The No. 3 formulation: 15% of FIR (Concn of 7%, FER of 12) and 6% of BIR (Concn of 6%);
4. The No. 4 formulation: 30% of FIR (Concn of 7%, FER of 12) and 6% of BIR (Concn of 6%);
5. The No. 5 formulation: 15% of FIR (Concn of 3%, FER of 12) and 6% of BIR (Concn of 10%);
6. The No. 6 formulation: 30% of FIR (Concn of 3%, FER of 12) and 6% of BIR (Concn of 10%).

**Fig. 13:**
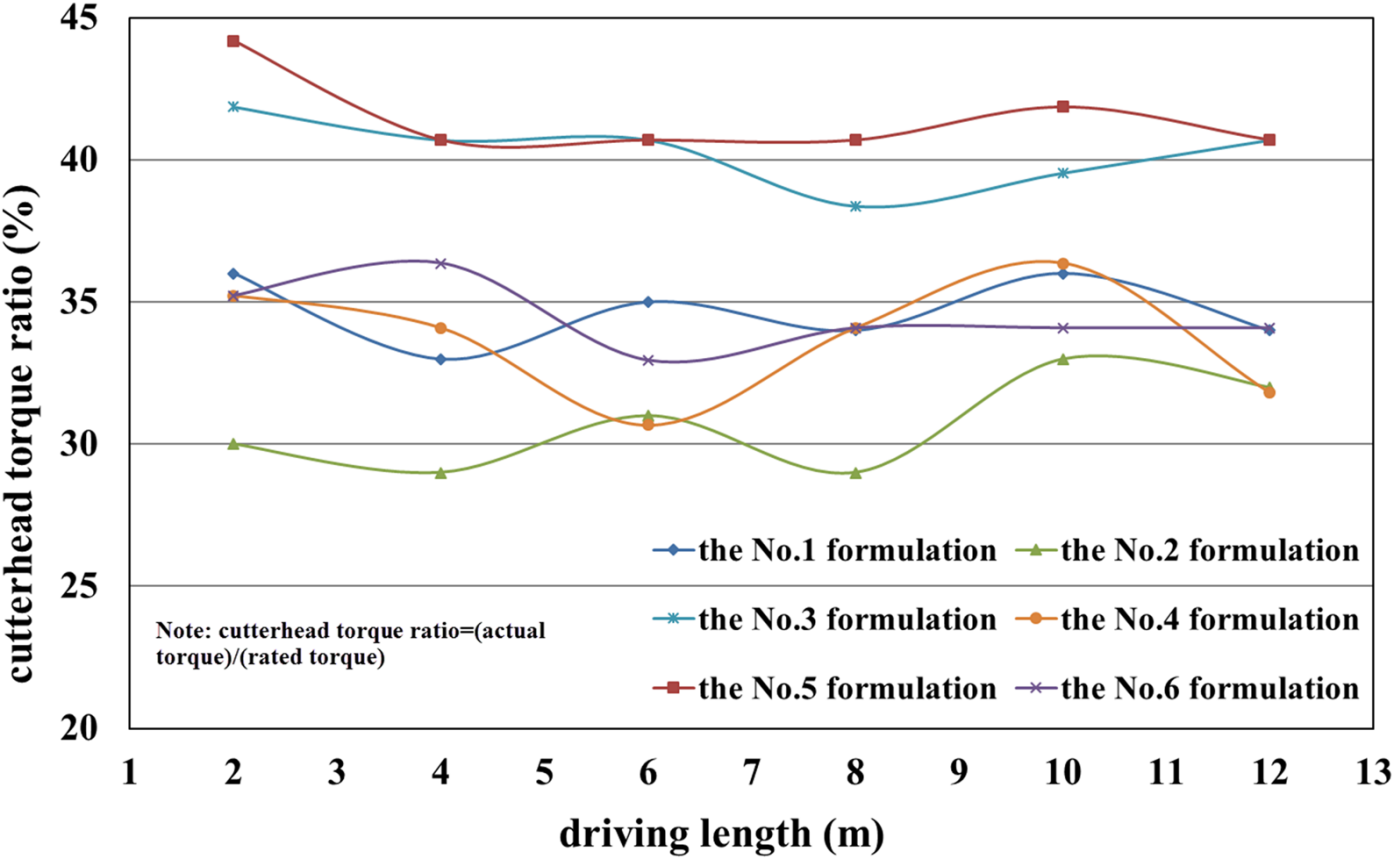
Recorded cutterhead torque

**Fig. 14:**
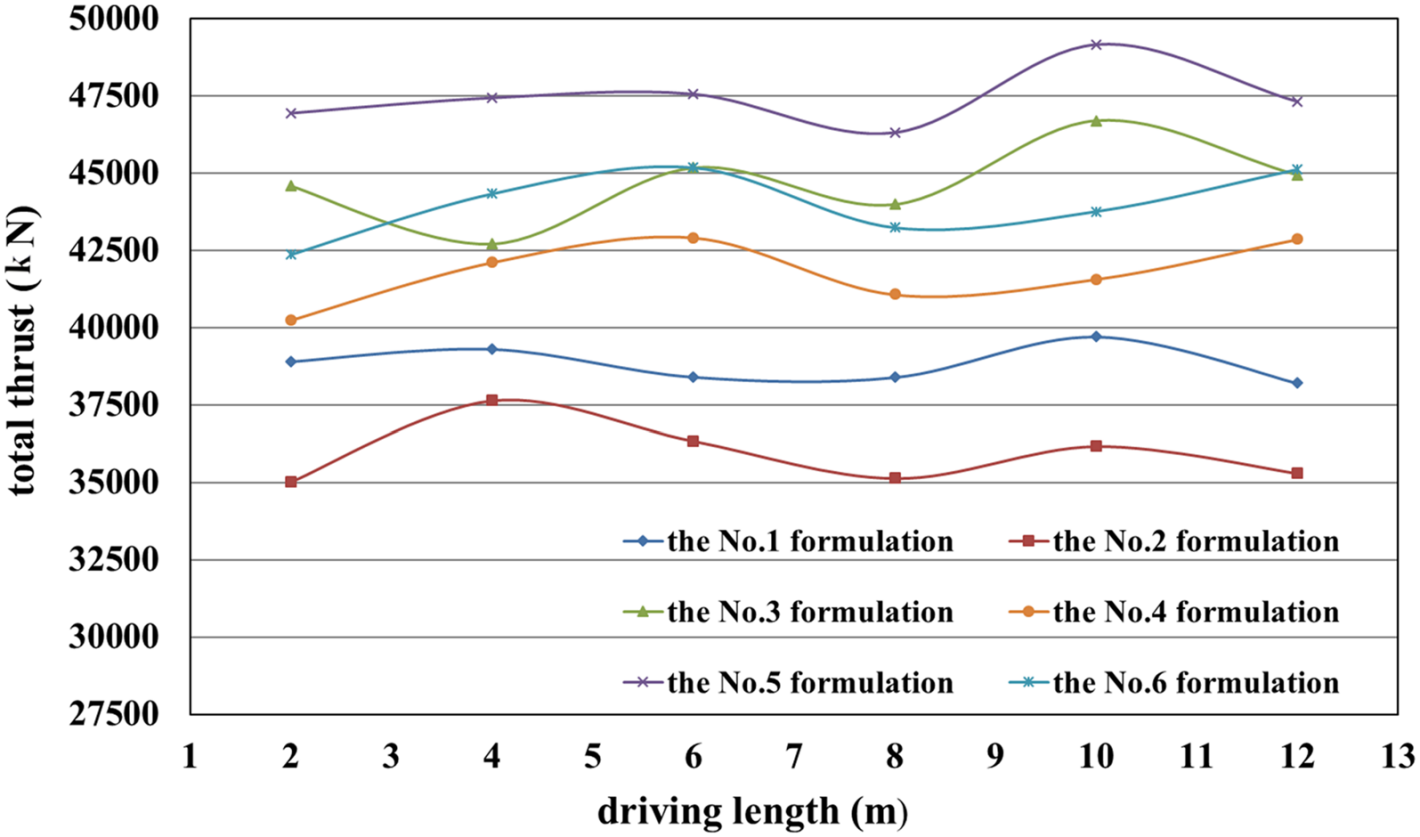
Recorded total thrust

**Fig. 15:**
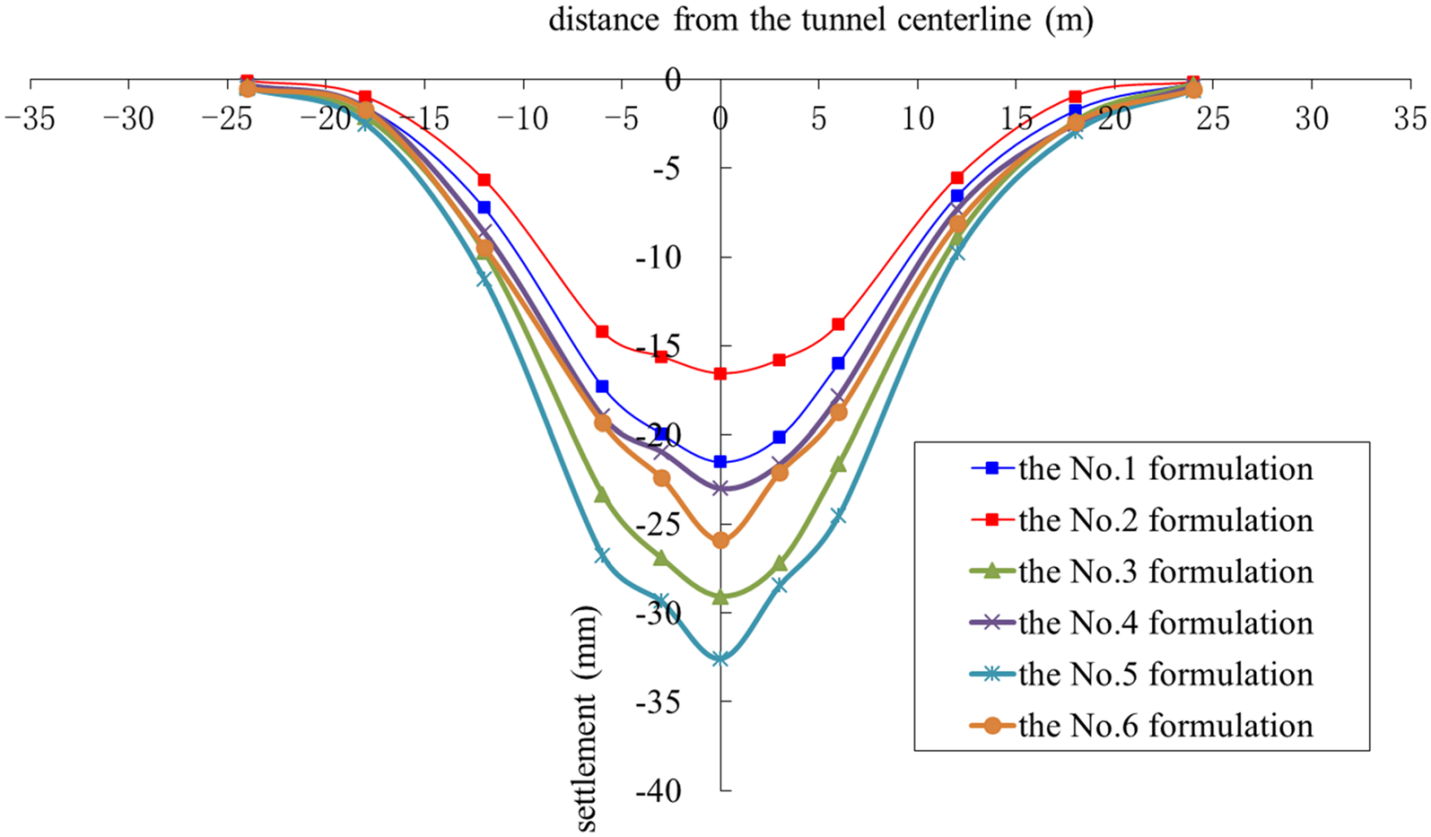
Measured ground surface settlements

In general, the well-controlled shield operation could be achieved when the slumps of the muck were more than 160 mm, with shield tunneling parameters remaining almost stable, as given in Figs. 13, 14 and 15. However, when the slumps reached 180 mm, the construction efficiency was significantly affected due to the difficulty in muck transport by the belt conveyor. Thus, the overly large slumps of the muck were not suitable. Higher earth chamber pressure and lower ground surface settlement were obtained when using the No. 1 and 2 formulations to condition the soil, as shown in Figs. 12 and 15. The surface settlement profiles in Fig. 15 were measured after grouting. When shield driving passing the test sections, the key shield operational parameter including grout injection volume were controlled at almost the same level to understand the conditioning effects using foam. The test sections were arranged with a spacing of more than 20 m, resulting the same influence of the test section locations on the measured settlements. Considering the ease of the operation, the conditioning scheme using the No. 1 formulation was suggested; however, the scheme using the No. 2 formulation was used, when more sand in the soils, with the increased injection ratio of the foam liquid. Variations of the total thrust and cutterhead torque with different formulations, as shown in Figs. 13 and 14, may have led to friction changes caused by different additives between the cutterhead, shield skin and soils.

### Grouting work

In practice, grouting of shield tunneling includes grouting of the tail void (first phase injection) and grouting directly through segments (second phase injection) (Guglielmetti et al. 2008). Based on previous experience in Beijing, attention was paid to the first phase injection, and a two-component grout was used in the injection.

The A-liquid mainly consisted of cement and bentonite (8% of Concn); the B-liquid consisted of sodium silicate (30 of Baume degree, specific gravity of about 1.26). To reduce the surface settlement to the largest degree, the coagulation time of the grout was controlled to be within 15–20 s by adjusting the mix ratios of the A-liquid to the B-liquid; meanwhile, an injection volume of 14.1–15.3 m^3^ was used in each segmental ring. The grout injection adopted was mainly based on the measured surface settlement.

#### Tests of the A-liquid

Sodium bentonite from two places of origin (Weifang of Shandong Province and Chengde of Hebei Province of China) and slurries of two concentrations were used to prepare the tests of the A-liquid. The recorded results are listed in Table 11. It is shown that the slurry with a Concn of 9% is prone to choking the injection device and the pipelines and is thus unfit for field applications. The market price of the A-liquid was also investigated, as shown in Table 12. In the some of the cells, two numbers mean materials were added twice and the plus-signs in the cells means that water was added twice. IST (Initial setting time) is the time at which grout paste loses its plasticity. After a comprehensive comparison, it was determined that bentonite slurry (yellowish white) 8% in Concn from Chengde of Hebei Province was used to prepare the A-liquid. The specific gravity of the slurry was 1.19 and the viscosity was 37.66 s.

**Table 11:** Test results of the A-liquid

Place of origin of the sodium bentonite	Mix ratio of the A-liquid in mass					Density (g/cm^3^)	Funnel discharge time (s)
	Water	Cement	Retarder	Concentration of the bentonite slurry			
				8%	9%		
Weifang of Shandong Province, red appearance	1	1	0.016	3.52		1.16	38.37
	1	1	0.016		3.13	1.19	115
Chengde of Hebei Province, yellowish white appearance	1	1	0.016	3.52		1.19	37.66
	1	1	0.016		3.13	1.20	65.84

**Table 12:** Market prices of the A-liquids of different mix ratios in China

Materials	Water	Cement	Slurry	Retarder	SG	IST (s)	TP (¥/m^3^)	Evaluation
1								
Mix ratio	1	1	0.3	0.013	1.32	16	209.75	The most reasonable
Mass (kg)	571 + 157.6	571	171.3	7.423				
	728.6		13.7					
Price (¥)	4.4	179.9	6.85	18.6				
2								
Mix ratio	1	0.9	0.3	0.01	1.27	35	188.64	Merits: low price; defects: long setting time and unfit for construction
Changed Mix ratio	1.11	1	0.33	0.0111				
Mass (kg)	574.7 + 158.6	517.19	172.40	5.747				
	733.3		13.8					
Price (¥)	4.47	162.9	6.9	14.37				
3								
Mix ratio	1	0.9	0.2	0.008	1.29	20	195.06	Merits: low price; defects: long setting time and unfit for construction
Changed Mix ratio	1.11	1	0.22	0.009				
Mass (kg)	611.95 + 112.6	550.76	122.39	4.896				
	724.6		9.79					
Price (¥)	4.42	173.5	4.9	12.24				
4								
Mix ratio	1	0.8	0.2	0.008	1.24	36	177.37	Merits: low price; defects: long setting time and unfit for construction
Changed Mix ratio	1.25	1	0.25	0.01				
Mass (kg)	617.53 + 113.63	494.02	123.51	4.94				
	731.16		9.88					
Price (¥)	4.46	155.62	4.94	12.35				

*SG* specific gravity, *IST* initial setting time, *TP* total price

#### Tests of mixing the A-liquid and B-liquid

Firstly, the A-liquid was prepared by mixing different materials, and then tests of mixing A-liquid and B-liquid were performed. The results of the tests are given in Table 13. In Table 13, Final setting time is the time when the paste completely loses its plasticity. It is the time taken for the grout paste to harden sufficiently.

**Table 13:** The results of the tests of mixing the A-liquids and B-liquids

Mix ratio in mass of the A-liquid				Density of A-liquid (g/cm^3^)	Mix ratio in volume of the A-liquid to the B-liquid	Final setting time (s)
Water	Cement	Slurry	Retarder			
0.8	1	0.3	0.013	1.31	10:1	20.5
1.1	1	0.22	0.009	1.29	10:1	20.2
1.1	1	0.23	0.013	1.32	10:1	23.5
1.1	1	0.33	0.011	1.27	10:1	35.2
1.25	1	0.25	0.010	1.24	10:1	36.3
1	1	0.25	0.013	1.32	10:1	16.5
1	1	0.3	0.013	1.32	10:1	16.4
1	1	0.4	0.013	1.32	10:1	17.3
1	1	0.5	0.013	1.32	10:1	25.9
1	1	0.6	0.013	1.32	10:1	35.2
1	1	3.52	0.016	1.21	10:1	24.6
1	1	3.52	0.016	1.21	11:1	25.6
1	1	3.52	0.016	1.21	12:1	28.7
1	1.2	3.52	0.016	1.22	12:1	14.7
1	1.4	3.52	0.016	1.23	12:1	13.5
1	1.08	0.37	0.012	1.32	10:1	17.1

#### Determining the mix ratio of the grout

The grout for backfilling the annulus gap should have a higher coagulation rate under the premise that the quick solidification of the grout does not choke the pipelines. After analyzing the test results and other data available, the solidification time of the grout was adopted to be 16.4 s, and the corresponding mix ratio of water:cement:slurry:retarder is 1:1:0.3:0.013.

**Fig. 16:**
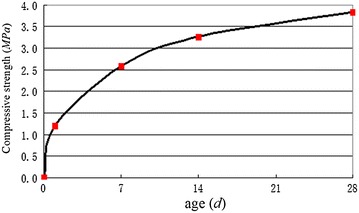
Variations with age of the compressive strength of the grout test block

Variations in the strength of the grout test block with age are given in Fig. 16, which is the test result of mixing the A-liquid and B-liquid. This type of grout is quickly solidified, with a rapid increase in strength, resulting in higher strength at 28 days, and quick or even immediate support to the surrounding ground.

## Performances of the shield machine

**Fig. 17:**
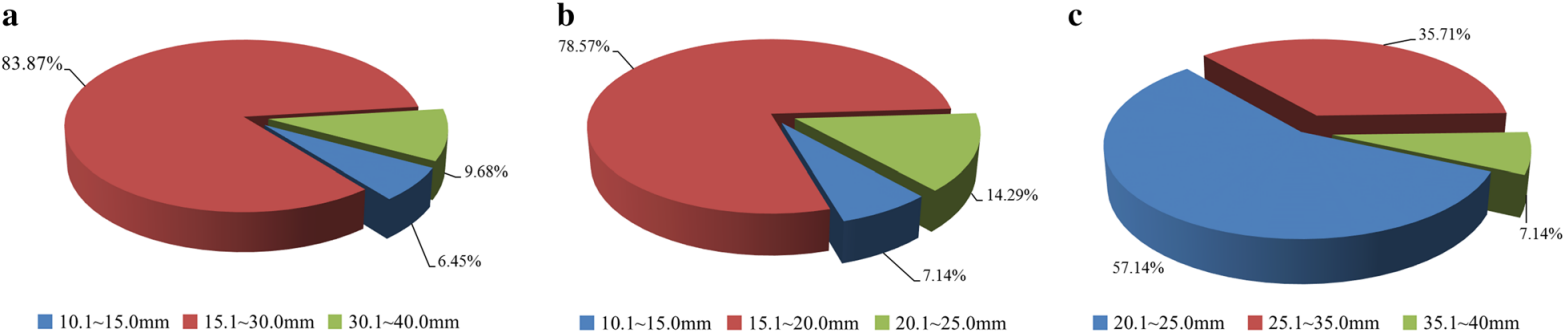
Measured maximum surface settlements. **a** All settlements, **b** clay soils at tunnel roof, **c** sandy soils at tunnel roof

During long distance driving of the shield, the scheme of simultaneously injecting foam and bentonite using the No. 1 formulation was usually adopted to control slumps of the excavated soils at approximately 160 mm. However, in difficult situations, such as passing large cross sections of sand soils or crucial risk sources, the injection scheme of the No. 2 formulation was used to increase the injection ratio of the foam. Compared with the No. 1 formulation, more foam was used in the No. 2 formulation. This led to the high earth chamber pressure, the less cutterhead torque and total thrust, and more importantly the small surface settlement. So the No. 2 formulation was used to get the small settlement in more difficult conditions. Generally, the above guidelines were executed in the whole tunnel construction, and the shield tunneling parameters were well regulated. The key parameters were as follows: earth pressure in the excavation chamber of approximately 0.12–0.18 MPa, total thrust of approximately 30,000–50,000 kN, cutterhead torque of approximately 30–45% of the rated torque, rotation of the cutterhead of approximately 0.4–0.7 rpm, and a shield advance rate of approximately 30–70 mm/min. While the active earth pressure balance was created at cutting face, the simultaneous backfilling of approximately 14.1–15.3 m^3^ per ring was exerted using the formulation from the field tests. The maximum ground surface settlements were well controlled within 10–40 mm, as shown in Fig. 17. The soils at the tunnel roof have a large influence on the surface settlement. When sand soils were encountered, the larger settlements of 20–40 mm were induced, as shown in Fig. 17c; when clay soils were encountered at the tunnel roof, the settlements were approximately 10–25 mm, as shown in Fig. 17b. As shown in Fig. 17a, most maximum surface settlements were 15.1–30 mm, accounting for 83.87% of the total. The percentage for each ground condition represents the distribution of the maximum surface settlement, which was the results of the shield tunneling operation (including the simultaneous backfilling grouting). A settlement of 40 mm was acceptable if no visible damages was done to the surroundings and structures. Surface settlements are also affected by many other factors, such as tunnel depth, shield operation parameters, injection pressure and volume (Banbendererde 1991; Bezuijen et al. 2004; Merritt and Mair 2008; Guo 2013).

As for buildings, houses, pipelines, main road and bridges in the vicinity, shield tunneling induced movements were strictly controlled below the predefined allowable movements with the auxiliary measures taken, such as grouting from the ground surface when necessary. Taking the shield under-crossing the viaduct of the Beijing Airport Express as an example (Guo and Li 2014), three dimensional numerical calculations were performed to predict the movements caused by the shield tunneling using the finite element method in advance. Based on the calculations, anchor-piles were used to reinforce the ground before shield under-crossing. During the under-crossing, strict control of the shield operation was executed, and the maximum settlement of the viaduct was <2 mm of the predefined allowable settlement. The settlement of 2 mm was required by the owner of Beijing Airport Express. Linear motor-driven trains were adopted on the Beijing Airport Express, which are sensitive to the settlement of the viaduct structure. To guarantee safety of the trains operation, the strict control criterion of 2 mm was required.

## Conclusions

Based on the application of the 10.22 m diameter EPB shield with a spoke-type cutterhead in Beijing subway construction, the following conclusions can be drawn:

1. Fluidity of the excavated soils, which are crucial to the creation of the earth pressure balance at the cutting face, can be achieved through two aspects of work. At first, using suitable additives is the key to condition the soils. The second aspect involves the full mixing of the additives and the excavated soils. For a shield more than 10 m in diameter, the second work is more important. The construction practice shows that the scheme of injecting the bentonite slurry (8% of Concn) and the foam liquid (5% of Concn) simultaneously to condition the excavated soils, the special designs of the 10.22 m diameter shield machine for the agitating system, the simultaneous backfilling grouting system and the independently driven agitating device are successful, and they can provide a useful reference for similar projects.
2. It is feasible for this shield of more than 10 m in diameter that slumps of the conditioned soils are controlled to approximately 160 mm. When a large cross section of sand or complex surface surroundings are encountered, more foam is required, and the injection ratio of the foam liquid should be increased.
3. The two-component system injection with the adopted mixing ratio of grout determined by the combination of indoor tests and field tests is reasonable for this diameter shield tunneling, resulting in maximum surface settlements of 15–40 mm.

## Abbreviations

EPB: earth pressure balance
Concn: concentration
BIR: bentonite injection ratio
FIR: foam injection ratios
FER: foam expansion ratios
SG: specific gravity
IST: initial setting time
TP: total price
